# Adulteration of Brandy

**Published:** 1883-07

**Authors:** 


### Adulteration of Brandy.

Some startling revelations have recently been made by the U. S. Consul at Rochelle regarding the quality of the liquor exported from France as brandy. He states that the whole business has become a gigantic fraud, and that little or no genuine brandy can be obtained, even by purchasers upon the spot. All the owners of large vineyards are distillers, and they are said to be heaping up colossal fortunes by manufacturing beet and potato spirit, which, by judicious diluting and flavoring, is made to resemble, in taste and appearance, brandy of any required age or make. When they sell brandy purporting to be twenty or thirty years old, or of a particular vintage, they simply mean that the article looks and tastes like genuine brandy of that stated age or vintage. The vile compounds with which the market is being flooded are highly injurious, not only from the irritating properties of fresh beet and potato spirit, but also from the poisonous qualities of the flavoring and coloring ingredients. As brandy ages, it loses its fiery properties, gains aroma and bouquet, and is then more suitable for medicinal purpose. Fresh new brandy should never be prescribed. It is therefore somewhat startling to learn that we cannot procure a genuine old brandy at any price, even from direct importers, and that we are probably prescribing for our patients the vile combinations of fraudulent French distillers instead of a bland and potent stimulant. If these corrupt practices are not speedily amended, the profession will have to seek elsewhere for a pure and reliable form of alcoholic stimulant, suitable for internal administration. —*Canada Medical Record.*

